# Long Term Evaluation of the Impact of Autologous Peripheral Blood Stem Cell Transplantation in Multiple Myeloma: A Cost-Effectiveness Analysis

**DOI:** 10.1371/journal.pone.0075047

**Published:** 2013-09-30

**Authors:** Alessandro Corso, Silvia Mangiacavalli, Federica Cocito, Cristiana Pascutto, Virginia Valeria Ferretti, Alessandra Pompa, Roberta Ciampichini, Lara Pochintesta, Lorenzo G. Mantovani

**Affiliations:** 1 Division of Hematology, Fondazione IRCCS Policlinico San Matteo, University of Pavia, Pavia, Italy; 2 Fondazione Charta, Milan, Italy; 3 Center of Pharmacoeconomics, Faculty of Pharmacy, University of Naples, Federico II, Italy; Centers for Disease Control and Prevention, United States of America

## Abstract

**Background:**

High-dose therapy with autologous peripheral stem cell transplantation represents today the standard approach for younger multiple myeloma patients. This study aimed to evaluate the long term economic impact of autologous transplantation with respect to conventional therapy.

**Methods:**

We retrospectively reviewed the charts of multiple myeloma patients diagnosed at our department between 1986 and 2003 and treated according to the therapy considered standard at the time of diagnosis. Analysis of costs was done by assessing resource utilization and direct costs were measured and monetized before proceeding with the analysis, based on public health service tariffs.

**Results:**

Group A including 78 patients treated with Melphalan and Prednisone was compared with Group B including 74 patients who received an autologous transplant. The median overall survival was 3.2 and 5.4 years respectively (p = 0.0002). Mean cost per patient was significantly higher in group B with respect to group A (102373€ vs 23825€; p<0.001). The final quality-adjusted-life-year gain in group B patients as compared to group A was 1.73 QALY, with an incremental cost-effectiveness ratio of 45460€. With a threshold of 75000€ per QALY gained, the cost effectiveness acceptability curve indicated that the probability that autologous transplantation in multiple myeloma is a cost-effective intervention is 90%.

**Conclusions:**

The cost of autologous transplantation remains high. The calculated incremental cost-effectiveness ratio, however, given the significant prolongation of overall survival obtained with autologous transplantation, is within an acceptable threshold. Notwithstanding, its high cost should be taken into account when considering the whole cost of multiple myeloma.

## Introduction

Multiple myeloma (MM) is a progressive hematologic malignancy accounting for approximately 0.8% of all cancer diagnoses and 0.9% of all cancer deaths worldwide [1]. During 1998–2002, it represented approximately 1.2% of all the cancers and 1.7% of all the cancer deaths in Italy.

Multiple myeloma is still a non-curable plasma cell neoplasm. Melphalan and prednisone (MP) has represented the backbone of therapy for more than 30 years in all settings of patients. At the beginning of the 90’s, two meta analyses [2], [3] of subsequent randomized trials, assessing more than 6000 patients, showed that outcomes are similar in patients treated with melphalan and prednisone or combination chemotherapy.

The use of high-dose chemotherapy with stem cell support has clearly improved disease-free survival and has increased overall survival of at least 24 months compared to the standard conventional chemotherapy MP regimen. As a consequence, patients’ quality of life has improved, also thanks to the better supportive therapies.

In the current health care environment, safety and efficacy are still the primary but no longer the only parameters to be evaluated to assess the value of a treatment. Costs and cost-effectiveness or cost-utility are becoming increasingly important. Moreover, health care systems are demanding health economic information as part of reimbursement submissions for new therapies.

Ageing and other causes of raising incidence of MM trigger a higher focus on disease management costs. A recent study focusing on this item has shown that the average social cost per year per patient is 76630€, for subject younger than 70 years old and 22892€ for the older group [4]. The higher costs deriving from the adoption of new treatment strategies have been balanced by the opportunity of a main out-patients managing with a positive impact in terms of survival and quality of life.

Although high-dose chemotherapy with stem cell support has been considered cost-effective compared to conventional chemotherapy, data reported in the literature are not univocal.

It is also still not clear whether the amelioration in the treatment of multiple myeloma with the adoption of this new strategy has improved other crucial outcomes related to disease management (e.g. decreasing the hospitalization rate and/or the necessity of ancillary therapies), producing in the same manner a significant decrease of MM management costs.

The intervention is associated with a substantial increase in quality adjusted life years (QALYs), given the prolongation of disease-free and overall survival time [5]. Quality adjusted life years are calculated from life expectancy by attributing a correction factor (between 0 and 1) to each life year which represents the patients’ evaluation of quality of life. Several studies have analyzed the cost-effectiveness of high-dose chemotherapy with stem cell support compared to conventional chemotherapy although such a threshold is likely to differ from one health care setting to another, and even from one disease area to another [5]–[11]. The ranges reported for the cost-effectiveness and cost-utility of high-dose chemotherapy with stem cell support compared to conventional chemotherapy may generally be regarded as acceptable, especially given the severity of the disease. Long-term economic assessments, however, are often either lacking, or facing methodological difficulties due to the limited availability of data and the need for extrapolations, which are or can be subject to assumptions.

Aim of this study was to perform a long term economic assessment by means of a cost-effectiveness analysis, of the impact of high-dose therapy on the overall survival of MM patients with respect to conventional chemotherapy.

## Methods

### Ethics Statement

The study was approved by the Ethics Committee of the Fondazione Istituto di Ricovero e Cura a Carattere Scientifico (IRCCS) Policlinico San Matteo, Pavia, Italy and conducted in accordance with the Declaration of Helsinki. The great part of patients was not able to sign a written informed consent because died. So, the consent was obtained by the approval of the ethic committee and of the privacy guarantor.

### Design and Patients

A monocentric longitudinal retrospective cohort of MM patients diagnosed between January 1st 1986 and December 31th 2003 and treated at the Hematology Department of Fondazione IRCCS Policlinico San Matteo (Pavia) was chosen as sample group of this study.

Patients were considered eligible for the study when having baseline characteristics suitable for transplantation whether or not high-dose therapy was an available treatment option at that time. High-dose melphalan with autologous stem cell transplantation in our Department has become a treatment option in 1995. Two groups of patients were therefore identified:

- Group A: patients receiving their first line of therapy between January 1^th^ 1986 and December 31^st^ 1994, never treated with autologous stem cell transplantation (patients who underwent a transplant later during the course of the disease were excluded from the study);
- Group B: patients receiving the first treatment between January 1^th^ 1995 and December 31^st^ 2003 with autologous stem cell transplantation.

Patient baseline characteristics (sex, age at 1^st^ treatment, MM type (IgG, IgG or Light chains), Karnofsky performance status, Durie and Salmon stage (I, II or III), presence of CRAB criteria (i.e. anemia, skeletal lesions (≥3, <3 vs No lesions), renal failure or hypercalcemia), survival data and therapeutic history were retrospectively collected from clinical records.

Analysis of costs was done by assessing resource utilization: for each hospital admission we collected: date, duration, cause, type of admission (out-patient visit, day-hospital, long-term hospitalization), type of administered therapies, type of concomitant treatments (particularly focusing on supportive therapies, i.e. transfusions, growth factors, antibiotics, biphosphonates). The direct costs for MM management were measured and monetized before proceeding with the analysis, based on public health service tariffs (Sistema sanitario regionale, SSR).

As the time span of the study would make a direct monetary comparison difficult, the current SSR tariffs were applied.

### Statistical Analysis

Continuous data were summarized by median and range; while categorical variables were summarized by relative frequency (%) in each category.

Inferential Statistics was applied to compare outcomes and costs between groups. The two-tailed Fisher’s exact test was applied to evaluate difference in the distribution of categorical variables between the two groups. The Mann-Whitney non-parametric test was used to compare numerical and ordinal variables between groups.

Overall survival (OS) curves were estimated using the Kaplan-Meier method and compared with the Gehan’s Wilcoxon test. The crude hazard ratio (HR) between the two groups was obtained from a Cox proportional hazards regression. An alpha (type 1 error) level of 0.05 was adopted for assessing significance. Microsoft Excel® and Microsoft Access® (© Microsoft) were used for data collection and management. Statistical analyses have been performed using Excel and Stata SE 11.2 (© Stata corp. LP).

### Cost-utility Analysis

A cost-utility analysis was carried out based on quality-adjusted life years (QALY). QALYs were computed by applying utilities to follow-up periods: 0.8 during off-treatment periods, 0.58 during therapy, and 0.63 during maintenance (values based on a literature review) [12], [13]. The incremental cost-effectiveness ratio (ICER) of treatment group B with respect to group A was calculated as the ratio between the incremental cost and the gain in QALY for a single patient.

In order to make the two groups comparable in terms of length of observation, 4 cases in group A were censored because they exceeded the maximum observation time in group B, i.e. 16.4 years. No correction was adopted to adjust for the fact that most patients are still being followed-up in group B, while all patients but two in group A have a complete follow-up. This might lead to an overestimate of the ICER in group B, because autologous stem -cell transplantation has the highest cost and occurs at the beginning of follow-up.

Uncertainty due to estimation of effects and costs was tested calculating the cost-effectiveness acceptability curve (CEAC) with the non-parametric bootstrapping approach [14]. A CEAC can be interpreted as the probability that an intervention is cost-effective compared with the alternative, given the observed data, for a range of maximum monetary values that a decision-maker might be willing to pay for a particular unit change in outcome.

## Results

### Patients Characteristics

Group A: 78 patients who received their first treatment between January 1^th^1986 and December 31^st^1994 plus four patients diagnosed after 1/1/1995 who never received autologous stem-cell transplantation.

Group B: 74 patients who received the first treatment between January 1^th^ 1995 and December 31^st^ 2003 and underwent autologous stem-cell transplantation.

Characteristics of patients are reported in table 1. In details, no difference was found between group regarding gender, age, myeloma type, CRAB criteria, baseline Karnofsky performance status. A significantly higher number of Durie & Salmon stage III patients was found in group B.

**Table 1:** Characteristics of patients.

	Group A (n = 78)	Group B (n = 74)	P
Gender (%: M/F)	60/40	50/50	0.25
Age at 1^st^ treatment (yrs: median, range)	55 (33–64)	53 (31–70)	0.35
Karnofsky (%: median, range)	80 (70–100)	85 (70–100)	0.26
Myeloma Type (%)			
IgG	66%	60%	
IgA	22%	19%	0.38
Light chains	12%	21%	
Durie & Salmon stage (%)			
I	30%	12%	
II	23%	18%	0.013
III	47%	70%	
Hemoglobin (g/dl: median, range)	10.9 (6–16.2)	10.9 (4.8–15.5)	0.59
Creatinine (mg/dl: median, range)	1 (0.3–6.2)	1 (0.6–4.9)	0.42
Calcium (mg/dl: median, range)	9.8 (8.6–15.6)	9.5 (8–17)	0.21
Skeletal lesions	47%	30%	0.048
Follow-up (years: median, range)	3.3 (0.2–21)	5 (0.1–16.4)	
Patients alive at the time of analysis	3%	24%	

**Figure 1:**
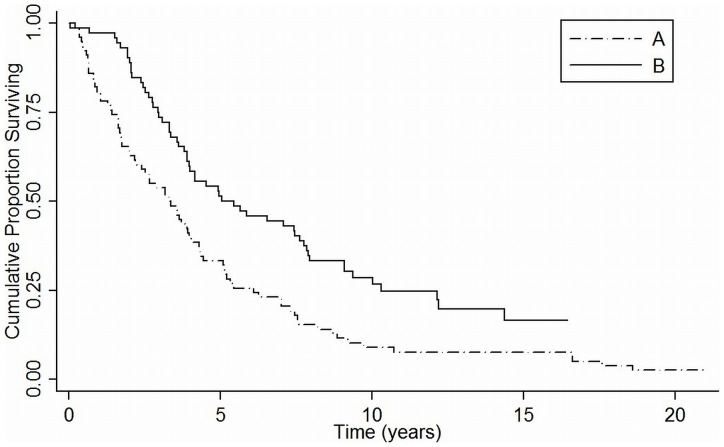
Overall survival (OS) in two groups. Better OS in the transplanted patients (group B)(p<0.001).

The final outcome distribution at time of last follow-up (august 2011) in the two groups was the following: patients alive in group A 3%, in group B 24%. After a median follow-up of 3.3 years and 5 years in group A and B, the median overall survival (OS) was 3.3 years and 5.4 years in group A vs B respectively, with a better outcome in transplanted patients (p = 0.0002, figure 1). The cumulative probability of survival at 1, 3, 5 years was respectively 80%, 53%, 33% and 97%, 73%, 51% in group A and B. The better survival in group B was confirmed by Cox regression: the estimated HR was 0.55 (95% CI 0.39–0.78, p = 0.001).

### Resources Utilization

A total number of 1756 hospital admissions were recorded, distributed as follows: 778 in group A and 875 in group B. The distribution of the causes of hospital admission is reported in table 2. Hospital admission distribution was different between the two groups (p<0.001), in particular we observed a lower rate of long-term hospitalizations in group A.

**Table 2:** Distribution of causes of hospital admissions.

Hospital Admission Type	Group A(n = 778)	Group B(n = 875)	P
Long term (%)	52%	73%	
• Treatment administration	58%	65%	
• Adverse events	27%	17%	<0.0001
• Transfusions	2%	1%	
• Other procedure	13%	17%	
Long term admissions/PYR	1.2	1.2	
Day hospital (%)	29%	13%	
• Treatment administration	56%	70%	
• Adverse events	–	–	0.07
• Transfusions	28%	16%	
• Other procedure	16%	14%	
Day hospital admissions/PYR	0.7	0.3	
Out-patient visit (%)	19%	14%	
• Treatment administration	82%	90%	
• Adverse events	–	–	0.08
• Transfusions	–	–	
• Other procedure	18%	10%	
Out-patient admissions/PYR	0.4	0.3	

Outpatient visits and day-hospital admissions were distributed as follows: 231 (29%) and 140 (19%) in group A and 243 (25%) and 128 (13%) in group B.

### Cost-utility

The mean cost per patient (including concomitant therapies) was significantly different between groups (p<0.001), with a single patient mean expense of 23825 € in group A vs 102373 € in group B. The total observation time was 336.4 and 478.2 person-years respectively in group A and B.

The observation time of each patient was subdivided into time-periods with different utilities: on therapy (utility = 0.58), on maintenance (utility = 0.63), not in treatment (i.e. in remission; utility = 0.8). The total quality of life-adjusted life years (QALYs) in the two groups were then calculated by applying the corresponding utility to each time-period.

When comparing Group B to group A, the gain in QALY per patient was 1.73 and, after accounting for the increase in cost, the resulting incremental cost-effectiveness ratio (ICER) was 45460 € per QALY gained. Details of the ICER computation are reported in Table 3.

**Table 3:** Details of incremental cost-effectiveness ratio computation.

	Group A	Group B	
Cost per person (€)	23824.5	102372.7	Incremental cost per person 78548.2 €
Total observation time (years)	336.4	478.2	
QALYs			Gain in QALY per person 1.73
• On therapy (utility: 0.58)	82.3	52.6	
• Maintenance (utility: 0.63)	9.2	42.2	ICER
• Off therapy (utility: 0.80)	143.9	256.4	45459.6 €
QALY/person	3.02	4.75	
Cost per autologous transplantation (€)		44454	ICER w/o transplantation13126.2 €

**Figure 2:**
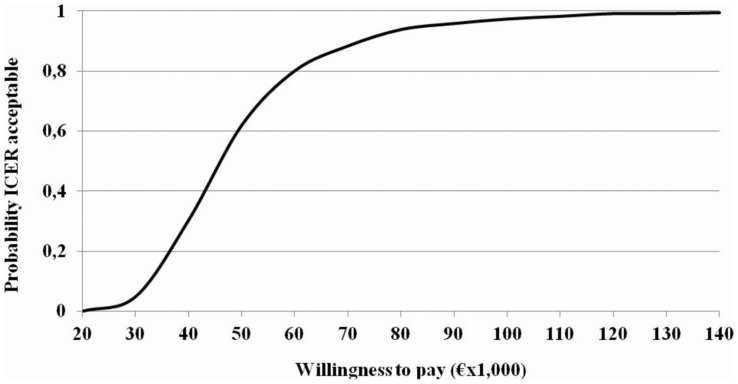
ICER acceptability curve.

The Cost effectiveness acceptability curve (Figure 2) indicated that autologous peripheral blood stem cell transplantation has more than 80% of probability of being cost effective at a threshold of 60.000 Euro per QALY gained, and more than 90% at a threshold of 75000 Euro per QALY gained.

## Discussion

This study compares all running costs relative to health care provided to two different groups of patients treated with conventional or high dose therapy including transplant in a single center in subsequent periods of time. All patients of both groups were eligible to receive a transplant as first line therapy, but this was performed only in patients of group 2 since at the time of diagnosis for patients of group 1 autologous transplant was not available. Transplant-related costs have been variably reported, and clearly different financing and accounting system and calculation methods make the comparison between different studies very difficult. Most authors of studies on this issue have estimated costs ranging between US $30000 and 50000 per QALY gained [5]–[11]. Kouroukis et al report an improvement in life expectancy over a 10-year observation period of 19.3 months and an incremental cost of $25710 Canadian per life-year gained [8]. Gullbrandsen et al. in its work assumed an increased survival time of 1.5 years, the gain in QALY was 1.2 and the incremental cost-utility was US $27000 per QALY [5]. Most of the published studies have a relatively short follow up and Moeremans et al. in a review underline that, from a longer term prospective, the cost-effectiveness of myeloablative treatment may be found more favorable [15]. In our study the improvement of life expectancy over a 16-year observation period was 2.1 years, the gain in QALY per patient was 1.73 and, after accounting for the increase in cost, the incremental cost-effectiveness ratio (ICER) was €45460 per QALY gained. It must be stressed that, in this study the period of observation was significantly longer than in the previous reports and no projections or assumptions were needed. In addition, at the best of our knowledge, this is the first study in which the costs relative to resource utilization were computed not only for the first line of therapy but for the whole course of the disease which was, actually, substantially different in the two groups of patients. This represents the power of this study, together with a very long term analysis and the bulk of data recorded, although a limit on data quality is posed by its retrospective nature.

The high cost of autologous transplantation (44454€ per single procedure) implies that, even after a very long term evaluation, the impact of this procedure with respect to conventional chemotherapy in terms of costs remains substantial.

The analysis of resource data, which regarded all the hospital admissions including type, duration, and reasons of the admissions, highlighted some differences between the two groups. Patients treated with conventional therapy showed a lower rate of long-term hospitalization with respect to those underwent to transplant but they showed a higher frequency of admissions for adverse events or complications. As far as the day hospital admissions were regarded, patients of group 1 had a higher need of admissions for supportive therapy. When analyzing the distribution of the time-periods in the two groups, patients of group 1 were under therapy for significantly longer periods than those in the transplant group. This means that quality of life is clearly better for patients treated with transplantation, which allows longer periods out of treatment and lower need of supportive care. Moreover, at the time of last follow up only 3% of patients were alive in group 1 and 24% in group 2, meaning that the advantage in terms of overall survival could still improve in patients submitted to transplant with respect to those treated with conventional therapy.

It should be considered that cost–effectiveness ratios do not themselves provide information about whether the treatment is a cost effective use of resources. This decision depends on the perspective of the health care payer. One approach often used to assess the value of a treatment is to compare its cost– effectiveness ratio with ratios obtained with treatments in other fields. Whether a more effective yet more expensive treatment is cost-effective depends on the health payer’s willingness to pay for additional benefits. The value of this threshold is difficult to quantify. In the last few years, a threshold of 60.000 Euro per QALY gained has been proposed for Italy [16]. Taking 60.000 Euro per QALY as the benchmark for an acceptable cost–effectiveness ratio in Italy, our point estimates indicate that autologous peripheral blood stem cell transplantation in multiple myeloma is on average a cost-effective intervention, and is likely to be so in 80% of cases, as indicated by the CEAC. Although no cost-effectiveness or cost-utility acceptability threshold for transplantation had been previously identified by any authority, and even considering the variability from one health care setting to another, an incremental cost-effectiveness ranging between Canadian $20000 and 100000 (approximately 15000 and 75000 Euro) per QALY has been considered acceptable given the severity of the disease [17]. If the latter figure is taken as a reference value, the CEAC indicates that the probability that autologous peripheral blood stem cell transplantation in multiple myeloma is a cost-effective intervention is approximately 90%.

In conclusion, this long term analysis shows that high-dose therapy with autologous peripheral stem cell transplantation leads to a significantly better overall survival, and to a relevant gain in terms of quality-adjusted life-years with respect to conventional therapy. Therefore, the average cost of €45460 per QALY, although apparently high, is acceptable since the substantial improvement of the outcome in such a severe disease. However, its high cost should be taken into account when considering the whole cost of multiple myeloma.

Future studies are needed to re-evaluate autologous transplant after the introduction of novel agents.
