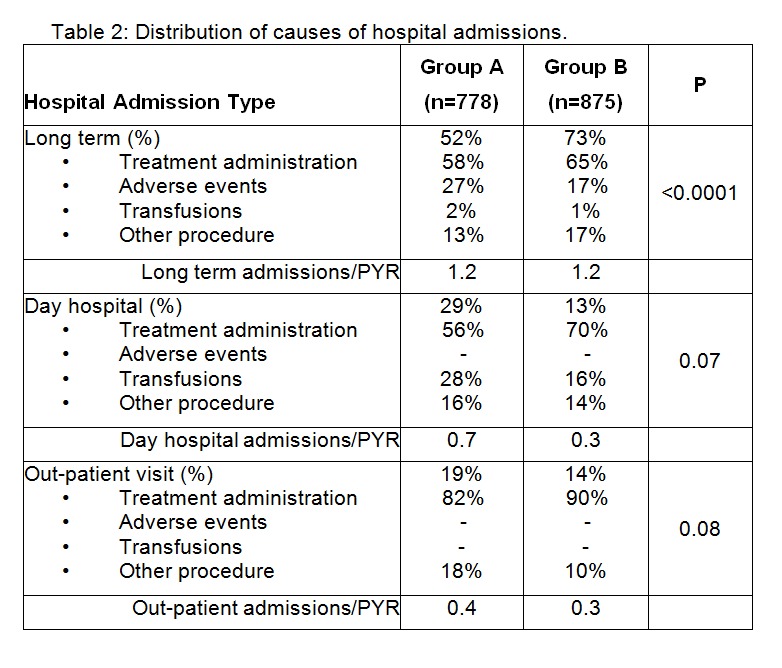# Correction: Long Term Evaluation of the Impact of Autologous Peripheral Blood Stem Cell Transplantation in Multiple Myeloma: A Cost-Effectiveness Analysis

**DOI:** 10.1371/annotation/949c27ad-65b0-4926-8c7b-5a45d67b1be9

**Published:** 2014-01-08

**Authors:** Alessandro Corso, Silvia Mangiacavalli, Federica Cocito, Cristiana Pascutto, Virginia Valeria Ferretti, Alessandra Pompa, Roberta Ciampichini, Lara Pochintesta, Lorenzo G. Mantovani

The three p-values in the far right column of Table 2 should align with the categories of Long term (%), Day hospital (%), and Out-patient visit (%). Please see the corrected Table 2 here: 

**Figure:**